# Suppressor mutations in the Glutamine Dumper1 protein dissociate disturbance in amino acid transport from other characteristics of the Gdu1D phenotype

**DOI:** 10.3389/fpls.2015.00593

**Published:** 2015-08-04

**Authors:** Shi Yu, Réjane Pratelli, Cynthia Denbow, Guillaume Pilot

**Affiliations:** Department of Plant Pathology, Physiology and Weed Science, Virginia Polytechnic Institute and State University, BlacksburgVA, USA

**Keywords:** *Arabidopsis*, suppressor screening, ethyl methanesulfonate, structure–function, amino acid transporter, glutamine transport

## Abstract

Intracellular amino acid transport across plant membranes is critical for metabolic pathways which are often split between different organelles. In addition, transport of amino acids across the plasma membrane enables the distribution of organic nitrogen through the saps between leaves and developing organs. Amino acid importers have been studied for more than two decades, and their role in this process is well-documented. While equally important, amino acid exporters are not well-characterized. The over-expression of *GDU1*, encoding a small membrane protein with one transmembrane domain, leads to enhancement of amino acid export by *Arabidopsis* cells, glutamine secretion at the leaf margin, early senescence and size reduction of the plant, possibly caused by the stimulation of amino acid exporter(s). Previous work reported the identification of suppressor mutations of the *GDU1* over-expression phenotype, which affected the *GDU1* and *LOG2* genes, the latter encoding a membrane-bound ubiquitin ligase interacting with GDU1. The present study focuses on the characterization of three additional suppressor mutations affecting *GDU1*. Size, phenotype, glutamine transport and amino acid tolerance were recorded for recapitulation plants and over-expressors of mutagenized GDU1 proteins. Unexpectedly, the over-expression of most mutated *GDU1* led to plants with enhanced amino acid export, but failing to display secretion of glutamine and size reduction. The results show that the various effects triggered by *GDU1* over-expression can be dissociated from one another by mutagenizing specific residues. The fact that these residues are not necessarily conserved suggests that the diverse biochemical properties of the GDU1 protein are not only born by the characterized transmembrane and VIMAG domains. These data provide a better understanding of the structure/function relationships of GDU1 and may enable modifying amino acid export in plants without detrimental effects on plant fitness.

## Introduction

Amino acids are critical metabolites in plants which fulfill several roles, in addition to being the constituting blocks of proteins. Amino acids are used as precursors for the synthesis of many secondary metabolites, like flavonoids (Falcone Ferreyra et al., 2012), alkaloids (Ziegler and Facchini, 2008), and glucosinolates (de Kraker and Gershenzon, 2011), which are metabolites critical for interaction of the plant with the environment (attraction, defense, and protection). Amino acids, especially Gln and Asn, also serve as essential carriers for organic nitrogen throughout the plant, being transported through the xylem and phloem saps between leaves, roots, storage organs, and meristems (Tegeder, 2014). Transport in the plant is mediated at the cell level by membrane proteins with specialized functions. Amino acid importers and exporters mediate transport of amino acids in opposite directions: importers mediate transport toward the cytosol, exporters mediate transport out from the cytosol, and respectively from or to the apoplasm, vacuoles or intracellular vesicles.

Characterized amino acid importers belong to the APC superfamily (Amino Acid Polyamine transporter; Vastermark et al., 2014), composed of 63 members in *Arabidopsis* (Tegeder and Rentsch, 2010). Importers utilize energy of the proton gradient across membranes to import amino acids against their concentration gradient, and are involved in many roles, like uptake from the soil, import into the phloem, phloem–xylem exchange, and transport into the embryo (Tegeder, 2014). Much less is known about amino acid exchangers and exporters. One member of the CAT subfamily has recently been described as an amino acid exchanger in tomato (Snowden et al., 2015). Two APC members have been described as possible amino acid exporters: CAT8 (Yang et al., 2010) and BAT1/GABP1 (Dundar and Bush, 2009; Michaeli et al., 2011). Finally, one gene belonging to the Drug and Metabolite Transporter superfamily (Jack et al., 2001), *SIAR1*, has been shown to unequivocally mediate amino acid export from plant cells (Ladwig et al., 2012). The family SIAR1 belongs to contains 47 members with only one other gene characterized, the auxin transporter WAT1 (Ranocha et al., 2013).

**FIGURE 1:**
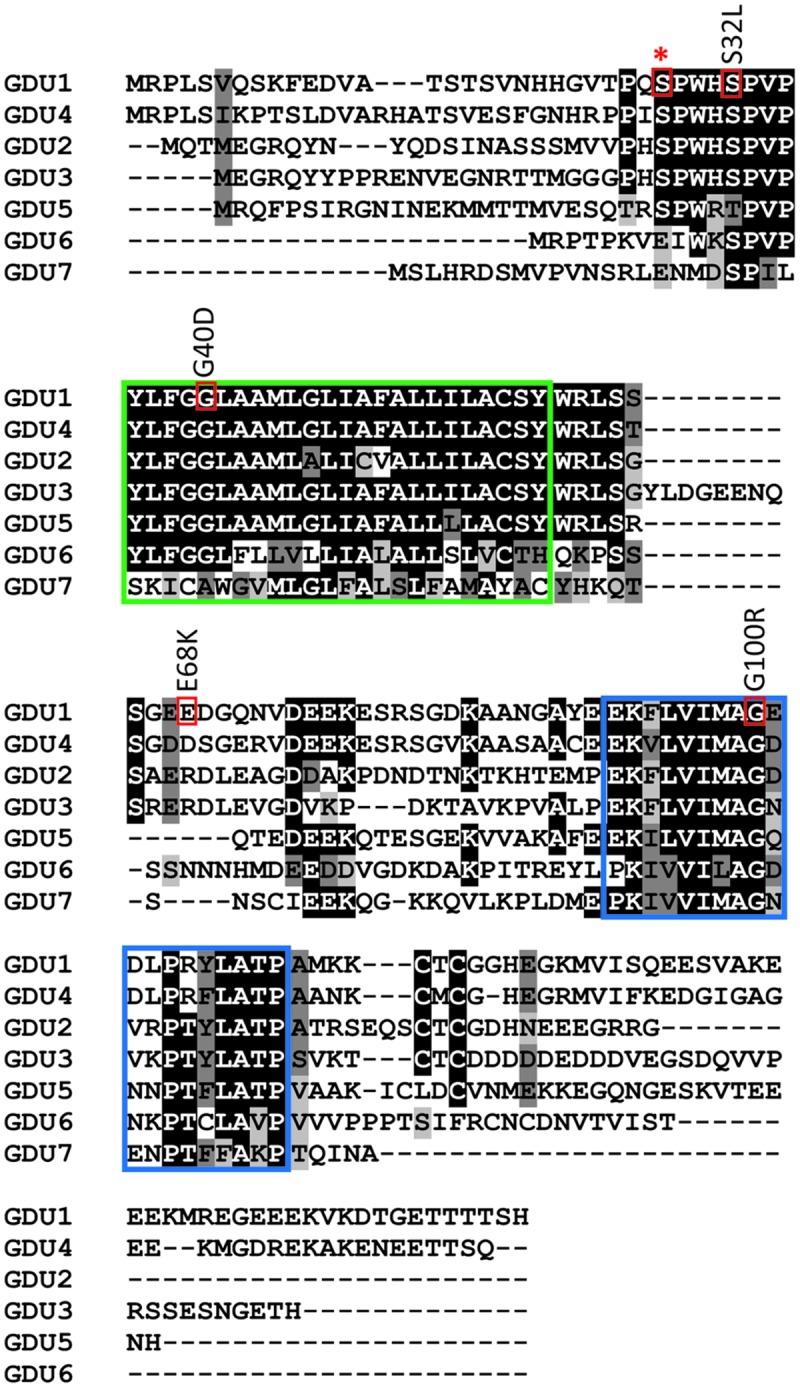
**Location of the *log1* suppressor mutations in GDU1.** Residues of interest are boxed in red, the conserved membrane and VIMAG domains are boxed in green and blue, respectively. Suppressor mutations are indicated above the corresponding boxes (the mutations in the *log1-1, log1-2, log1-3*, and *log1-4* lines correspond to G100R, E68K, G40D, S32L respectively in the protein sequence). Star indicates GDU1 S28, a predicted phosphorylation site located on the extracellular side of GDU1.

The existence of processes controlling the activity of amino acid transporters was evidenced by the discovery of the *GDU1* gene, encoding an 158 amino acid protein, with a single transmembrane domain (Pilot et al., 2004). Two domains are conserved among GDU proteins: a membrane domain, and a cytosolic 19 amino acid-long region, called the VIMAG domain (Pilot et al., 2004; see **Figure 1**). Over-expression of *GDU1*, for instance in the *gdu1-1D* mutant, leads to a complex phenotype characterized by reduced plant size; early leaf senescence; crystallization of Gln at the leaf margins; increased amino acid content in leaves, apoplasm, and xylem and phloem saps; tolerance to exogenously supplied amino acids; and notably enhanced amino acid export from cells, while amino acid import remains unaffected (Pilot et al., 2004; Pratelli and Pilot, 2007; Pratelli et al., 2010). This complex phenotype (called Gdu1D) can almost entirely be explained by enhanced amino acid export from cells: this phenomenon would increase amino acid content in the apoplasm and phloem and xylem saps, and prevent absorption of Gln from the xylem in the leaf, which then is excreted by the hydathodes (Pilot et al., 2004). Size reduction likely comes from the induced disturbance in nitrogen metabolism. The fact that over-expression of *GDU1* in *Nicotiana tabacum* and *GDU1*-homologs in *Arabidopsis* leads to a Gdu1D-similar phenotype suggests that the proteins of this family have a conserved function in plants, related to the regulation of amino acid export (Pratelli and Pilot, 2006; Pratelli et al., 2010). Nevertheless, the precise function of GDU1 in this process remains unknown.

Attempts to understand the function of GDU1 in the plant led to the identification of the ubiquitin ligase LOG2 in a yeast-two-hybrid screening. GDU1 and LOG2 localize at the plasma membrane and are able to co-immunoprecipitate when expressed in *Nicotiana benthamiana* (Pratelli et al., 2012). Decrease in *LOG2* expression suppressed the Gdu1D phenotype, indicating that LOG2 activity is necessary for the development of the phenotype (Pratelli et al., 2012). A mammalian homolog of LOG2 is the mahogunin protein (MGRN1; 45% similarity at the protein level), involved in the regulation of the activity and trafficking of membrane proteins and in degrading aggregated proteins (Jiao et al., 2009; Perez-Oliva et al., 2009; Gunn et al., 2013; Chhangani et al., 2014). Despite coming from different organisms, MGRN1 and LOG2 were found to have several overlapping functional properties, notably the ability of the mammalian protein to partially complement the loss of the plant LOG2 in *Arabidopsis* (Guerra et al., 2013). It is hypothesized that LOG2 and GDU1 are involved in the regulation of the activity or trafficking of amino acid exporters, such as, when *GDU1* is over-expressed, the exporters are more active at the plasma membrane (Pratelli et al., 2012; Guerra et al., 2013).

An ethyl methanesulfonate (EMS) screening was previously performed to isolate suppressor mutations of the Gdu1D phenotype. For this purpose, two *GDU1* over-expressing lines (*gdu1-5D* and *gdu1-6D*, created by introducing a *GDU1*-over-expressing construct into wild type plants) were EMS-mutagenized, and screened for progenies that grew similarly to the wild type. These suppressor lines hence over-express a mutant GDU1 protein in addition to the endogenous GDU1 protein. Two suppressor mutants, *loss of gdu1-1* and *2-1* (*log1-1* and *log2-1*) were isolated and characterized (Pratelli and Pilot, 2006; Pratelli et al., 2012). *log1-1* carries a G100R substitution in the conserved VIMAG domain of GDU1, which abolishes the interaction with LOG2 (Pratelli et al., 2012). The *log2-1* is a R12K substitution in LOG2, whose effect has not been determined at the biochemical level (Pratelli et al., 2012). Another mutation, *log2-3*, was isolated from the same screening, and is a nonsense mutation in LOG2 (R15stop; Pratelli and Pilot, unpublished data). These three *log* mutations led to plants that were phenotypically indistinguishable from the wild type when grown on soil and on amino acid-containing media. From the same screening, we isolated three additional *log1* mutations, whose characterization is reported here.

## Results

### Identification of Three New Mutations Suppressing the Gdu1D Phenotype

The pipeline previously described by Pratelli and Pilot (2006) was used to isolate new Gdu1D suppressor mutants: visual screening of M2 plants from the EMS mutagenesis of the *GDU1* over-expressors *gdu1-5D* and *gdu1-6D*, and confirmation of continued *GDU1* mRNA over-accumulation. Out of a total of 110,000 M2 seeds screened, three additional suppressor mutations were isolated that nearly restored the wild type size of the plants (Supplementary Figure S1), and suppressed the early senescence and Gln secretion. Analysis of the phenotype of F2 plants from crosses of the mutants with *gdu1-6D* and the wild type Col-7 showed that the three new mutations were recessive and segregated in accordance with an intragenic mutation in *GDU1* (data not shown). Similar to *log1-1*, the corresponding mutants came from the mutagenesis of the over-expressor *gdu1-6D* (Pratelli and Pilot, 2006), and were named *log1-2, log1-3*, and *log1-4*. Sequencing of the *GDU1* CDS in the T-DNA construct leading to its over-expression revealed that the mutations in *log1-2, log1-3*, and *log1-4* corresponded to G202A, G119A, and C95T mutations (numbered from the ATG), respectively, in the *GDU1* DNA sequence. These mutations led to E68K, G40D, and S32L substitutions, respectively, in the GDU1 protein sequence (**Figure 1**). Because these mutations suppressed the Gdu1D visual phenotype (plant size, Gln secretion, and early senescence), it was hypothesized that they affected GDU1 protein function. The *log1* mutants were hence hypothesized to over-express inactive GDU1 proteins and the characterization of the mutations was thus a way to better understand GDU1’s structure–function relationships.

**FIGURE 2:**
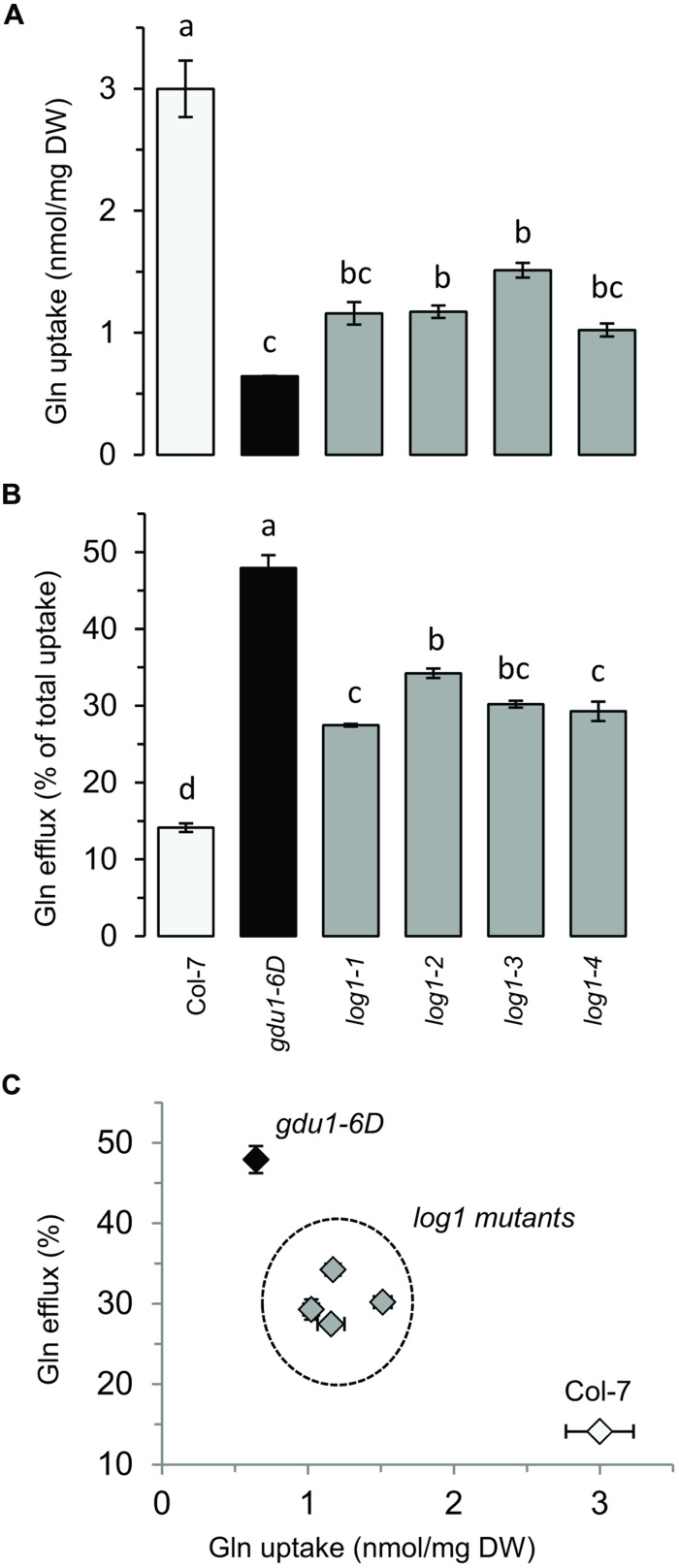
**Gln uptake and eﬄux analyses of the *log1* suppressor mutants. (A)** Gln uptake and **(B)** Gln eﬄux of 2 week-old plantlets. Eﬄux is expressed as the percentage of total Gln uptake. **(C)** Graph constructed by compiling the results of **(A,B)**. The wild type Col-7 and *gdu1-6D* are indicated as white and black bars and symbols respectively. Error bars are SEM (*n* = 3); values with same letters are not statistically different, as determined by ANOVA using Tukey’s HSD, *p* < 0.05.

Amino acid uptake of *GDU1* over-expressing lines was shown to be reduced, while eﬄux was enhanced (Pratelli et al., 2010). It was expected that the suppressor lines would display similar uptake and eﬄux as wild type plants, because of their wild type phenotype on soil. To test this hypothesis, Gln uptake of the suppressor lines was analyzed and compared to the wild type and the parental line *gdu1-6D*. Since this assay was not performed in the *log1-1* plants (Pratelli and Pilot, 2006), they were included in the present study. The *log1-1, log1-2, log1-3*, and *log1-4* suppressor mutants showed uptake and eﬄux similar to one another, but surprisingly different from both the wild type and *gdu1-6D*: the four *log1* mutants had an uptake about 60% lower than the wild type (**Figure 2A**) and an eﬄux twice as large as the wild type (**Figure 2B**). As a comparison, Gln uptake of *gdu1-6D* was 75% lower and the eﬄux was three times larger than the wild type. The intermediate phenotype was clearly visible when uptake and eﬄux were plotted on the same graph (**Figure 2C**). This result suggests that these recessive suppressor mutations decrease GDU1 overall activity and thus lead to a milder phenotype. It has indeed been shown that the strength of the Gdu1D phenotype depends on the *GDU1* expression level and that the plants are small and display early senescing leaves only when GDU1 is over-expressed 10 times or more than the wild type (Pilot et al., 2004).

### Characterization of Plants Over-Expressing the GDU1 Variant Proteins

**FIGURE 3:**
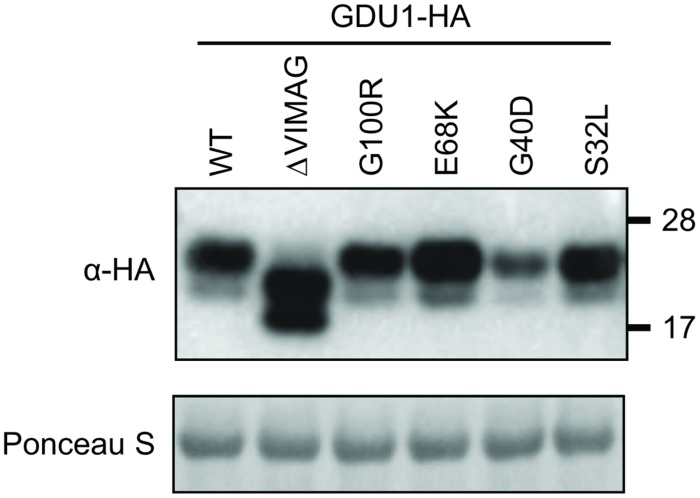
**Accumulation of the GDU1 protein variants in *N. benthamiana* leaves.** Accumulation of the HA-tagged GDU1 protein variants was estimated by western blot with an anti-HA antibody. The same amount of *Agrobacterium* was infiltrated per leaf. WT: wild type; ΔVIMAG: GDU1 protein with the VIMAG domain deleted. Expected sizes: GDU1 and *LOG* suppressor variants, ∼21.8 kDa; ΔVIMAG GDU1, 19.8 kDa. GDU1 has always been found to migrate as two bands on a western blot (see Pratelli et al., 2012).

Despite two different attempts, no suitable antibody could be raised against GDU1. The *gdu1-5D* and *gdu1-6D* plants and the corresponding *log* suppressor mutants express an un-tagged GDU1 protein, preventing any quantitation of the GDU1 protein accumulation. In order to test for any effect of the suppressor mutations on the accumulation of the GDU1 protein, wild type GDU1, the three GDU1 variants, G100R GDU1 (corresponding to the *log1-1* suppressor mutant; Pratelli and Pilot, 2006) and a GDU1 protein lacking the VIMAG domain (ΔVIMAG) were fused with the HA tag, placed under the control of the CaMV 35S promoter, and expressed in *N. benthamiana*. The ΔVIMAG, G100R, E68K, and S32L mutations did not affect protein accumulation, while the G40D mutation led to a consistent reduction of about 30% in protein accumulation in this system (**Figure 3**). The sub-cellular localization of these five GDU1 variants was determined by expression of the GFP-tagged proteins (GFP positioned in C-terminal) in *N. benthamiana* leaves and observed by confocal microscopy. Similar to GDU1, the proteins localized to the plasma membrane and in compartments that could correspond to endosomes, similar to the wild type GDU1 protein (Pratelli et al., 2012). Interestingly, the fluorescence of these compartments was stronger for the ΔVIMAG, G100R and G40D GDU1 proteins, suggesting that the proteins located more in these structures than the other GDU1 proteins (Supplementary Figure S2), possibly revealing some effect of the mutations on the protein properties.

**FIGURE 4:**
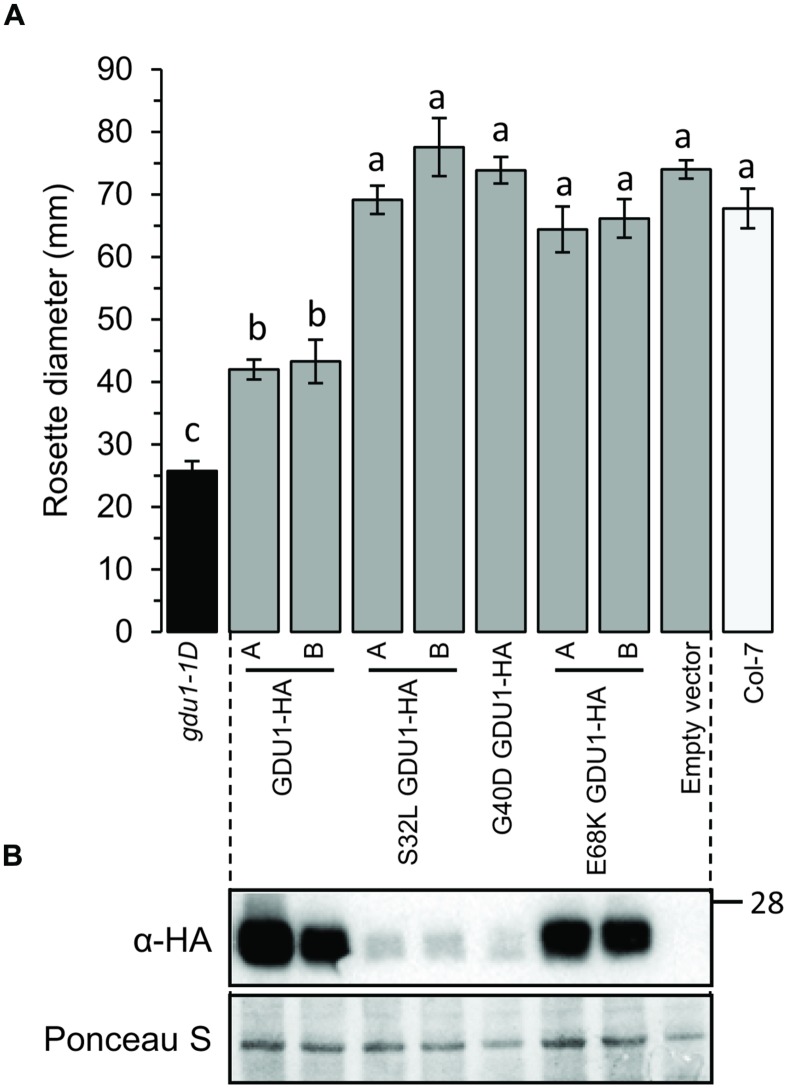
**Analysis of plants over-expressing the *GDU1* variants. (A)** Rosette diameter of 4 week-old *Arabidopsis*. Error bars are SEM of 6–8 plants; statistical significance determined by ANOVA using Tukey’s HSD *p* < 0.01. **(B)** Accumulation of the HA-tagged GDU1 proteins in each line was estimated by western blot with an anti-HA antibody. The wild type Col-7 and *gdu1-1D* lines are indicated as white and black bars; these lines do not express any tagged protein and were not tested by western blotting.

To study the ability of the proteins to lead to the Gdu1D phenotype, the HA-fusion constructs were used to transform *Arabidopsis* and create recapitulation lines. The accumulation of the proteins in lines that segregated 3:1 for the kanamycin resistance was tested by western blot (**Figure 4B**). G40D GDU1-HA accumulated at a lower level than GDU1-HA, in good agreement with the *N. benthamiana* results. Surprisingly, S32L GDU1-HA did not accumulate to the GDU1-HA levels in both tested lines, but was still present at high levels. In all cases, the rosette sizes of the plants expressing the GDU1 suppressor variants were identical to the wild type. The plants over-expressing GDU1-HA showed a ∼45% reduction in rosette diameter compared to the wild type and the empty vector-transformed plants (**Figure 4A**). Only the GDU1-HA over-expressors displayed Gln secretion crystals, typical of the Gdu1D phenotype. The size reduction of the GDU1-HA lines was less than for the original *gdu1-1D* over-expressor (∼60%; **Figure 4A**), and could be attributed to the difference in the construct used or the presence of the HA tag, which might slightly interfere with the protein stability or activity.

**FIGURE 5:**
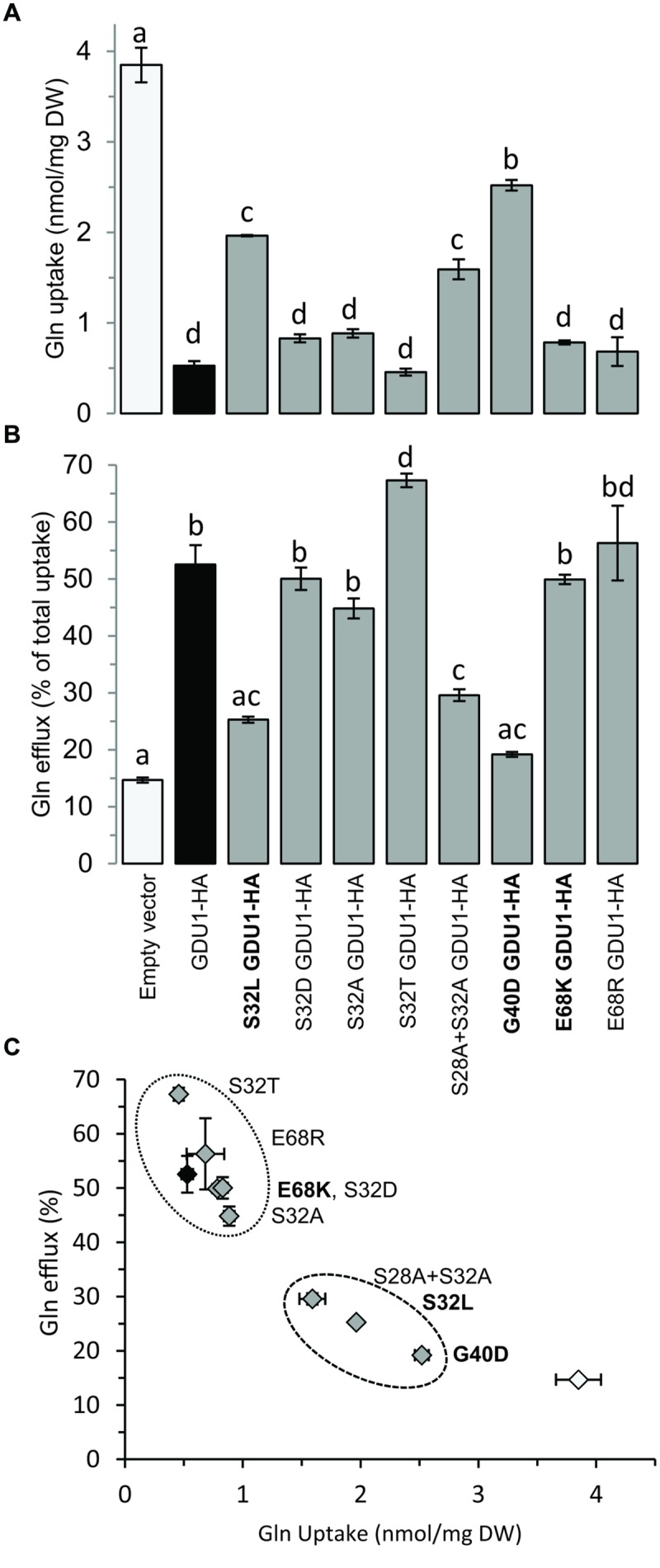
**Gln uptake and eﬄux analyses of plants over-expressing the *GDU1* variants. (A)** Gln uptake and **(B)** Gln eﬄux of 2 week-old plantlets. Eﬄux is expressed as a percentage of total Gln uptake. **(C)** Graph compiling the results of **(A,B)**. Recapitulation lines to the *log1-2, log1-3*, and *log1-4* mutants are indicated in bold; the empty vector- and *GDU1*-over-expressing lines are indicated as white and black bars and symbols respectively. Error bars are SEM (*n* = 3); statistical significance determined by ANOVA using Tukey’s HSD *p* < 0.05.

Since the *log1* mutants showed intermediate Gln uptake and eﬄux between the wild type and the *gdu1-6D* mutant, Gln transport by the recapitulation lines was studied. G40D and S32L GDU1-HA over-expressing plants behaved similarly to the corresponding *log1-3* and *log1-4* suppressor mutants: the uptake was reduced by ∼50% while the eﬄux was increased by ∼50–100%. On the contrary, E68K GDU1-HA over-expressors transported Gln similarly to the GDU1-HA plants, differently from the *log1-2* plants that they were supposed to recapitulate (**Figures 5A,B**). This discrepancy between the Gln uptake and eﬄux of the *log1-2* suppressor mutant and E68K GDU1-HA over-expressors was unexpected and is not completely explained. It is possible that the presence of the HA tag interferes with the function of the mutated protein, similar to what was observed with the wild type GDU1 (the GDU1-HA over-expression line exhibits a weaker phenotype than the original *gdu1-1D*; **Figure 4A**). These data prove that the intermediate phenotype of the *log1-3* and *log1-4* mutants is caused by the over-expression of variant GDU1 proteins endowed with reduced functionality.

### Testing the GDU1 Variants for Interaction with LOG2

The suppression of the Gdu1D phenotype by the G100R mutation (in the *log1-1* mutant; Pratelli and Pilot, 2006) was explained by the loss of interaction with LOG2, the ubiquitin ligase necessary for the development of the Gdu1D phenotype (Pratelli et al., 2012). The G100R mutation affects the Gly100 residue of the VIMAG domain that is conserved in all GDU proteins examined so far. This Gly to Arg mutation (**Figure 1**) is supposed to either affect the folding of the VIMAG domain or create a steric clash at the interface surface between GDU1 and LOG2 caused by changes in residue charge and/or size. Because the three other *log1* mutants displayed a similar phenotype as *log1-1*, we tested if any of the corresponding mutations would affect the GDU1-LOG2 interaction, which would explain the suppression of the Gdu1D phenotype.

**FIGURE 6:**
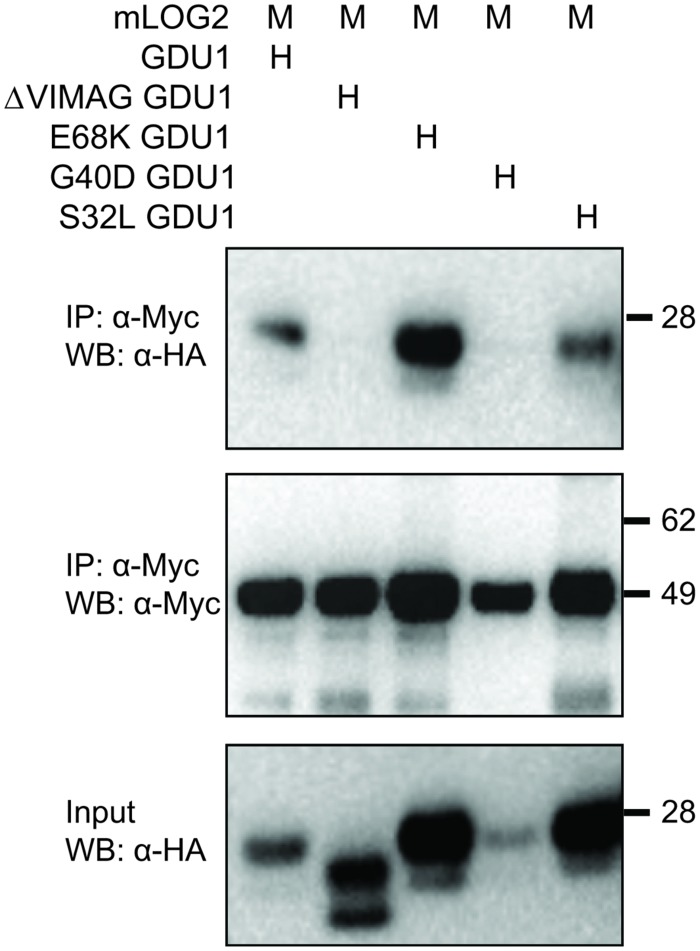
**Co-immunoprecipitation of the GDU1 variants and mLOG2 expressed in *N. benthamiana*.** GDU1 variants were expressed as C-terminal HA fusion (H), mLOG2 (ubiquitination defective) as C-terminal Myc fusions (M) in *N. benthamiana* leaves. Proteins were extracted and immunoprecipitated (IP) with an anti-cMyc antibody, and detected by western blot (WB). Numbers on the right indicate molecular weight in kDa. In this particular experiment, LOG2-Myc (lane 4) did not accumulate at the same level as in the other samples.

The LOG2 and GDU1 proteins were fused with the Myc or the HA tag respectively, expressed in *N. benthamiana* leaves and immunoprecipitated using cMyc agarose beads. The ΔVIMAG GDU1 protein, shown to be unable to interact with LOG2 (Pratelli et al., 2012) and to lead to the Gdu1D phenotype when over-expressed (Pratelli and Pilot, 2006), was used as a negative control (**Figure 6**). GDU1-HA, E68K GDU1-HA, and S32L GDU1-HA could be co-purified with LOG2-Myc, but G40D GDU1-HA did not co-immunoprecipitate (**Figure 6**). The G40D mutation, in addition to decreasing protein abundance (**Figure 3**), thus seems to prevent the interaction of GDU1 with LOG2, which could explain the Gdu1D suppressor effect. On the contrary, the Gdu1D suppressor effect of the other variants cannot be explained by an inability to interact with LOG2.

### Site Directed Mutagenesis to Understand the S32L Mutation

Ser32 is highly conserved among GDU proteins, being sometimes replaced by Thr only, a residue with similar chemical properties (in 13% of the ∼100 analyzed sequences from 20 plant species, including lower plants, conifers, monocots, and dicots; data not shown). Running prediction algorithms such as PhosPhAT (Heazlewood et al., 2008; Durek et al., 2010) and PlantPhos (Lee et al., 2011) suggested that Ser32 can be phosphorylated (Supplementary Figure S3) despite the fact that this part of the protein is supposed to be extra-cellular. The PhosPhAT tool also predicted that Ser28, next to Ser32, can be phosphorylated. We hypothesized that these two Ser can be phosphorylated, and that this phosphorylation is important for GDU1 function. Ser32 was mutagenized to Thr, Asp, and Ala to test for (1) the importance of the Ser vs. Thr in GDU1 function, the effect of (2) mimicking and (3) suppressing phosphorylation at this site respectively. Ser28 and Ser32 were also mutagenized to Ala at the same time to ensure that lack of Ser32 phosphorylation could not be complemented by phosphorylation of Ser28. The mutagenized GDU1 proteins were stably expressed in *Arabidopsis* in fusion with the HA tag. The size of the plants, protein accumulation, and Gln transport were determined as above.

**FIGURE 7:**
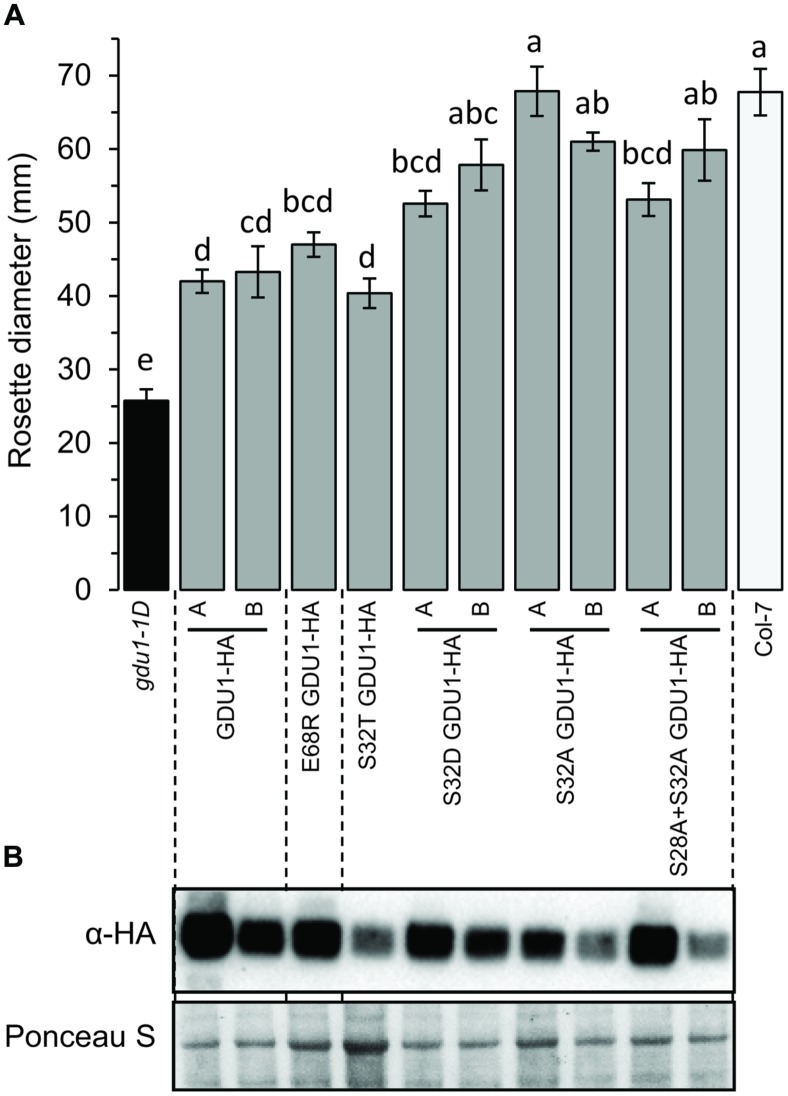
**Analysis of plants over-expressing the GDU1 variants. (A)** Rosette diameter of 4 week-old *Arabidopsis*. Plants were grown at the same time as plants in **Figure 1** (the values for *gdu1-1D*, GDU1-HA and Col-7 are the same as in **Figure 1**). Error bars are SEM of 6–8 plants; statistical significance determined by ANOVA using Tukey’s HSD *p* < 0.01. **(B)** Accumulation of the HA-tagged GDU1 proteins in each line was estimated by western blot with an anti-HA antibody. The wild type Col-7 and *gdu1-1D* lines are indicated as white and black bars; these lines do not express any tagged protein and were not tested by western blotting.

**FIGURE 8:**
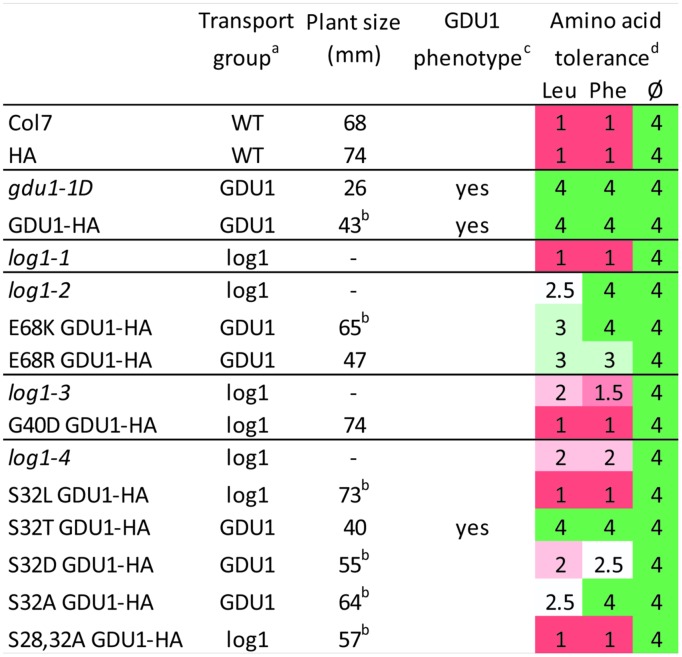
**Summary of uptake, size, phenotype, and amino acid tolerance of the *log1* suppressor mutants and of lines over-expressing the GDU1 variants.** ^a^WT: uptake and eﬄux similar to the wild type; GDU1: uptake and eﬄux similar to *gdu1-1D*; log1: intermediate between *gdu1-1D* and the wild type (see **Figures 2** and **5**). ^b^Value is the average of two lines (see **Figures 4** and **7**); “-” indicates that the line was not grown in this experiment. ^c^Gln secretion and early senescence. ^d^Growth was scored according to the growth of the wild type (set to 1) and the *gdu1-1D* mutant (set to 4) in the corresponding growth condition; wild type and gdu1-1D plants grew equally well on the medium lacking amino acids (Ø); average of two experiments (the pictures of the plants from one experiment are displayed in Supplementary Figure S4).

Western blotting confirmed that all lines expressed the GDU1 protein variants (**Figure 7B**). The size and the phenotype of the plants over-expressing S32T GDU1 was identical to the GDU1-HA over-expressors, in terms of size, Gln secretion and leaf senescence (**Figures 7A** and **8**), suggesting that S32T GDU1 is fully functional. Over-expression of the S32D, S32A, and S28A+S32A mutant proteins led to plants with slightly reduced size (down to 80% of the wild type), but never as much as the GDU1 or S32T GDU1 over-expressors. On the other hand, Gln transport analyses showed that the plants over-expressing the S32D, S32A, and S32T variants displayed uptake and eﬄux similar to the plants over-expressing the wild type GDU1 (**Figure 5C**). It is worth noting plants over-expressing the S32T variant displayed a higher eﬄux than the GDU1-HA over-expressor (**Figure 5B**), suggesting that this protein is more active than GDU1. Interestingly, when the GDU1 protein bearing the double mutation S28A+S32A was over-expressed, Gln uptake and eﬄux were very similar to the S32L mutation, present in the *log1-4* mutant. No difference between the effect of the S32A and S32D mutations was detected by this assay.

### A E68R Mutation Leads to an Almost Fully Functional GDU1

We tested whether the suppressor effect of the E68K mutation came from the change in charge (negative to positive) or from the specific substitution to a Lys residue. For this purpose, Glu68 was mutagenized to Arg (a positively charged amino acid), and the variant protein expressed in *Arabidopsis*. This protein accumulated at the same level as the wild type protein in the over-expression lines (**Figure 7B**), and the corresponding plants had a size similar to one over-expressing the wild type *GDU1* (**Figure 7A**) but did not display the Gdu1D phenotype, i.e., secretion and early senescence (**Figure 8**). Transport assays showed that the plants over-expressing E68R behaved essentially similar to a *GDU1* over-expressor (**Figure 5**), showing that the Arg at this position has little effect on the protein function in this assay.

### Analysis of Amino Acid Tolerance of the Suppressed Mutants and of Over-Expressors of the GDU1 Variants

Some amino acids, when supplied at high concentration, have been shown to inhibit cell and plant growth (Bonner et al., 1992; Lee et al., 2007; Pratelli and Pilot, 2007).

Another characteristic of the Gdu1D phenotype is the tolerance of *GDU1* over-expressors to toxic concentrations of amino acids (Pratelli and Pilot, 2007; Pratelli et al., 2010). The effect of high concentration of amino acids was used as a supplemental assay to characterize the phenotype of the *log1* suppressors, the recapitulation lines and the over-expressors of the *GDU1* variants. The presence, absence or strength of the Gdu1D phenotype was tested by growing the plants on 10 mM Leu and Phe, shown to be particularly toxic to wild type plants (Pratelli and Pilot, 2007). All lines grew equally on the control medium (**Figure 8** and Supplementary Figure S4), but showed remarkable differences on the Leu- and Phe-supplemented media. The *log1-1, log1-3*, and *log1-4* mutants behaved similarly to the wild type, while *log1-2* behaved intermediately between *gdu1-1D* and the wild type, with strong tolerance on Phe but not Leu. Both E68K and E68R GDU1 over-expressors were fairly tolerant to Leu and Phe, in good accordance with the Gln transport assays, reminiscent of the Gdu1D phenotype. The recapitulation line over-expressing G40D GDU1 was as sensitive as the wild type. The S32L and S28A+3S2A GDU1 over-expressors behaved similarly to the wild type; the S32T GDU1 over-expressor looked like the *gdu1-1D* mutant; and the S32D and S32A mutants showed an intermediate phenotype between the wild type and the *gdu1-1D* mutant (**Figure 8** and Supplementary Figure S4).

## Discussion

### Suppressed Mutants Show Only Partially Abolished Gln Uptake

A total of four mutants were found (*log1-1, log1-2, log1-3*, and *log1-4*), all carrying recessive mutations, hence expected to behave as loss-of-functions, that suppressed the Gdu1D phenotype caused by the over-expression of the GDU1 protein. These suppressor lines over-express a mutant GDU1 protein in addition to the endogenous GDU1 protein. The thorough characterization of these lines presented in this study led to unexpected observations. The size of all the mutants was similar to the wild type and the early senescence observed in the leaves of *gdu1-1D* and *gdu1-6D* was absent. However, analysis of Gln uptake showed that the mutations did not suppress the Gdu1D phenotype completely (**Figure 2**), with uptake and eﬄux of the mutants being intermediate between the wild type and the parental line *gdu1-6D*. Interestingly, the suppressor mutation in *log2-1*, which affects the ubiquitin ligase LOG2, interactor of GDU1 (Pratelli et al., 2012), similarly does not completely suppress all characteristics of the Gdu1D phenotype: the wild type size is restored, the amino acid susceptibility is abolished, the plants do not secrete Gln anymore, but amino acid export is lowered to an intermediate level between the wild type and the parent (Pratelli and Pilot, unpublished data).

These mutations were obtained from a visual screening which led to the isolation of non-secreting plants of wild type size, possibly introducing a bias toward finding mutations that not necessarily affect transport, but rather toward ones that affect plant size and Gln secretion. The fact that the mutations in the *log1-1, log1-3*, and *log2* suppressor mutants abolish the Gdu1D phenotype partially, in a similar way suggest that they suppress the same component of the phenotype, e.g., the one involving LOG2, leaving other components intact. This implies that the over-expression of *GDU1* leads to several independent effects, some characterized by decreased size, induction of early senescence and amino acid tolerance, and others characterized by increased amino acid export.

The exact role of GDU1 is not known but two proteins interacting with it have been identified: LOG2 and its homolog LUL1, two membrane-associated ubiquitin ligases (Pratelli et al., 2012). It is proposed that GDU1 and LOG2/LUL1 form a complex stable enough to be co-immunoprecipitated, and involved in ubiquitination of yet unknown target(s) with a role in the regulation of amino acid transport (Pratelli et al., 2012). Loss of interaction with, or loss of expression of LOG2 is a simple explanation for the absence of the Gdu1D phenotype in the *log1-1* and *log1-3* mutants, over-expressors of a VIMAG domain-deleted GDU1 (Pratelli and Pilot, 2006) or the *log2* mutants (Pratelli et al., 2012). The fact that the loss of this interaction leads to a similar phenotype as the *log1-2* and *log1-4* mutants is striking, since the corresponding mutations in GDU1 (S32L and E68K) do not appear to affect the interaction with LOG2 (**Figure 6**). However, it is possible that the functional properties of the GDU1-LOG2 complexes resulting from the interaction of S32L and E68K GDU1 and LOG2 are affected by these mutations, explaining the resulting phenotype of the corresponding plants.

The active structure of GDU1 is not known, and a possible structure could be a multimer, either with itself or other GDU proteins (seven GDU proteins are present in the *Arabidopsis* genome). In this case, the mutated proteins in the suppressor mutants might assemble with the endogenous wild type proteins to form hetero-multimers, which could have retained only some functions of the original multimers while being less active. This hypothesis could explain the attenuated phenotype of the plants, in terms of uptake, size or amino acid tolerance, and the diversity of the phenotypes of the plants over-expressing the various GDU1 variants (**Figure 8**). It has then to be postulated that GDU1 has diverse functions, and that each suppressor mutation affects them differently. These functions remain to be determined.

### The G40D Mutation Might Affect GDU1 Protein Stability and/or Function

The G40D suppressor mutation in GDU1 has two effects on the protein’s properties: it does not accumulate at the same level as the other proteins when expressed in *N. benthamiana* leaves (**Figure 3**), and it does not co-immunoprecipitate with LOG2 (**Figure 6**). This mutation is located in the transmembrane domain, and affects one of the conserved residues of this domain. In about 100 GDU proteins from higher plants, Gly40 is sometimes replaced by Ala (8%), Ser (4%), Leu (1%), or Val (1%), but never by Asp (data not shown). Gly is a very common residue in membrane helices, involved in the formation of a glycine zipper in helix packing, thus enabling helix–helix interactions (Javadpour et al., 1999; Kim et al., 2005). Mutations of such important Gly residues led to abolition of transport (G114A in DctA from *Sinorhizobium meliloti;* Trainer et al., 2007) or loss of dimer interaction in EmrE from *Escherichia coli* (Elbaz et al., 2008), supposedly by disruption of the helix structure or ability to interact. The amount of sequence conservation of the GDU helix across different species is rare for a membrane protein, and one explanation could be that it is involved in interaction with membrane helices from other proteins. In this context, the G40D mutation likely affects such interaction, and may be the reason for the Gdu1D suppressor effect on the Gdu1D phenotype. Alternatively, the presence of a charged residue (Asp) inside the hydrophobic membrane helix could destabilize the protein, making it more difficult to be integrated in the lipid bilayer. This could explain the lower accumulation of the protein: membrane proteins that have folding problems are typically degraded by the cellular quality control system (Nagy and Sanders, 2004; Houck and Cyr, 2012). While the localization of the G40D GDU1 protein was not dramatically changed in *N. benthamiana* leaves, this does not preclude that GDU1 and its variants are addressed to different membrane subdomains. Such a hypothesis is supported by the fact that G40D GDU1 does not interact with LOG2: these proteins would not interact if G40D GDU1 and LOG2 are targeted to different membrane subdomains. Changes in subcellular distribution have indeed been observed for mutations affecting transmembrane Gly residues (Rosnoblet et al., 2013). Nevertheless, the fact that the over-expression of G40D GDU1 in wild type plants affects amino acid transport (**Figure 5C**) supports the idea that the protein still retained at least one of its functions, the one affecting amino acid transport.

### Putative Role of the External Ser28 and Ser32

The suppressor mutation in *log1-4* affected Ser32, which is predicted to be phosphorylated, together with Ser28 (Supplementary Figure S3). It is not clear if these residues can be phosphorylated *in vivo*, because they are expected to lie in the extracellular region of the GDU1 protein. Based on the identification of *bona fide* extracellular phosphoproteins with no transmembrane domain by a proteomic analysis of the extracellular matrix of *Arabidopsis* cell suspension cultures, an extracellular phosphorylation network has been suggested (Ndimba et al., 2003). ATP has been found in the extracellular medium, and is supposed to be involved in phosphorylation of extracellular proteins, as well as in intercellular signaling or during pathogen recognition (Ndimba et al., 2003; Chivasa et al., 2005). The fact that the mutation of the conserved Ser32 to Leu suppresses the Gdu1D phenotype led to the hypothesis that phosphorylation is important in GDU1 function. To test this hypothesis, Ser32 was mutagenized to Asp and Ala (to mimic or suppress phosphorylation, respectively) or Thr (another phosphorylation site), and over-expressed in plants. None of these mutations affected the functional properties of the GDU1 proteins as related to its effects on amino acid transport, since the over-expressors displayed similar Gln transport as GDU1 over-expressors (**Figure 5**). On the contrary, while S32T GDU1 over-expressors were small and tolerated amino acids, S32A and S32D over-expressors had near wild type size (**Figure 7**) and were somewhat susceptible to amino acids, suggesting that the S32T mutation does not alter GDU1 functional properties. S32A and S32D thus dissociated Gln uptake/export from the size and amino acid tolerance (**Figure 8**), showing that there is no direct relationship between these characteristics of the phenotype. The fact that their effect is similar is unexpected since these mutations typically lead to opposite phenotypes in terms of phosphorylation.

The double S28A+S32A mutation created a protein with functional properties very similar to the suppressor S32L mutation, and similar to the *log1* mutants (**Figures 2, 5, 7**, and **8**), different from the single S32A mutation. Ser28 and Ser32 thus seem to have redundant functions. In the hypothesis that these Ser are extra-cellular phosphorylation sites, mutagenesis of Ser32 individually would not have any effect, as observed; and only mutagenesis of the two Ser at the same time would lead to a phenotype. This hypothesis could be tested by mutagenizing Ser28 to Asp and Ala, which is expected to lead to a similar phenotype as the S32D and S32A over-expressing plants respectively. It can also be tested by mutagenizing S28 and S32 to Asp at the same time, the expected outcome of which would be a hyperactive GDU1 protein, and lead to plants with a phenotype reverse of the S28A+S32A over-expressing plants. In this hypothesis, the S32L mutation would prevent the phosphorylation of Ser28 and Ser32, possibly by affecting binding to a protein kinase, and would explain the observed similarity of the effect of S32L with S28A+S32A. If these Ser are not phosphorylation sites, these residues have a role in the function of the protein, which will require further investigation.

### Substitution of Glu68 by a Positive Residue Affects GDU1 Protein Function

The effect of the suppressor mutation E68K has been investigated based on the hypothesis that it locally changes the charge of the protein from negative to positive. The E68R mutation was expected to lead to a non-functional GDU1, which, when expressed in plants, would lead to the same effect as the E68K GDU1 from the *log1-2* mutant. Plants over-expressing E68R GDU1 were smaller, and Gln transport was similar to the *GDU1* over-expressors, suggesting that this variant GDU1 protein retained much of GDU1 functionality. The main difference with the *gdu1-1D* over-expressor is that these plants did not secrete Gln or display early senescence.

Surprisingly, the phenotype of the recapitulation line E68K GDU1 did not recapitulate the *log1-2* suppressor mutant. Amino acid transport in these plants was similar to the *gdu1-1D* mutant, and not intermediate between this mutant and the wild type as expected from the analysis of the *log1-2* suppressor mutant (**Figures 2** and **5**). The E68K GDU1 over-expressing plants displayed a phenotype similar to the plants over-expressing the E68R mutant, except that the size of the plants was similar to the wild type. It is not explained why the E68K GDU1-HA over-expressors do not behave as the *log1-2* plants and will need further investigation. As suggested earlier, the addition of an HA tag to the protein could affect the function of the mutant protein and explain this discrepancy. It has also to be noted that the *log1-2* mutant is tolerant to external amino acids, similar to the *gdu1-1D* mutant (**Figure 8**), but does not display size reduction, Gln secretion or early senescence.

These results suggest that the mutation of Glu68 to Lys, a positively charged residue, affects protein function in a way that the over-expressing plants display enhanced Gln transport but no dramatic growth alterations. Glu68 is not conserved in GDU sequences, and is sometimes replaced by Arg in GDU2 and GDU3 (**Figure 1**). Yet GDU2 and GDU3 over-expressors display the typical Gdu1D phenotype (small plants secreting Gln; Pratelli et al., 2010), showing that the function of the GDU2 and GDU3 proteins is not very different from GDU1, despite this difference in sequence.

### Conclusion and Opportunities for Crop Engineering

This work showed that all specific mutations in GDU1 can dissociate the various components of the Gdu1D phenotype. In some cases, the amino acid transport is affected in plants over-expressing mutant GDU1 proteins as in *gdu1-1D*, but these plants grow similarly to the wild type (S32A, E68K, and S32D). In the other cases, the transport is less affected, and the plants show a wild type phenotype (G40D, S32L) or close to wild type phenotype (S28A+S32A). The residues important for each function of GDU1 are not necessarily born by conserved residues, or domains, like the membrane or VIMAG domains. The use of the wild type GDU1 protein as a tool to modify amino acid export was impaired by the associated effects on plant fitness in *Arabidopsis* (Pilot et al., 2004) and tobacco (Pratelli and Pilot, 2006). The mutant proteins described here could be used to modify amino acid transport, and in particular export, in the plant without affecting plant fitness. Targeting amino acid export in plants would be another strategy to modify allocation of amino acids between the plant organs, and ultimately control protein content in reserve organs, like seeds and roots.

## Materials and Methods

### Plant Material and Growth

*Arabidopsis thaliana* (ecotype Col-7) lines were grown under 120 μE/m^2^/s, 22°C, 16 h light/8 h dark on soil (Mix of Sunshine Mix 1 and Pro-mix HP at a 1:1 ratio) and were watered from below with 300 mg/l Miracle-Gro Fertilizer [24/8/16% (w/w) N/P/K; Scotts, Marysville, OH, USA]. Rosette diameter of about eight plants from each line was measured with a ruler about 4 weeks after sowing. *Arabidopsis thaliana* were transformed by the floral dip method (Clough and Bent, 1998) using *Agrobacterium tumefaciens* GV3101 (pMP90). For transient expression of proteins in *N. benthamiana*, young leaves of 5-week-old plants were infiltrated with a suspension of *Agrobacterium tumefaciens* carrying the constructs of interest and the silencing suppressor p19 (Voinnet et al., 2003) according to (Batoko et al., 2000), with the following modifications. The bacteria were grown overnight in LB supplemented with appropriate antibiotics, washed twice in 10 mM MgCl_2_, 100 μM acetosyringone, and diluted to final OD_600_ of 0.05 in the same solution before infiltration in *N. benthamiana* leaves. Amino acid tolerance experiments were performed as described (Pratelli and Pilot, 2006): plants were grown for 10 days in long days conditions, on half-strength MS medium, supplemented with 0.5% sucrose, and 10 mM of indicated amino acids.

### Cloning and Constructs

Primer sequences used for cloning are listed in Supplementary Table S1. The *log1* variants of the *GDU1* gene were cloned by PCR from genomic DNA and cloned into pDONR Zeo by the Gateway technology. The Kunkel method was used for site directed mutagenesis (Kunkel et al., 1991), from sequences cloned by Gateway cloning in the pDONR Zeo Gateway vector, containing the f1 replication origin (Lalonde et al., 2010). Mutagenized inserts were sequenced, and transferred by Gateway cloning (Life Technologies) to the binary vector pPWHTkan and pPWGTkan, derivative of pJHA212K (Yoo et al., 2004; Pratelli and Pilot, unpublished data). pPWHTkan carries in this order the CaMV 35S promoter, the Gateway cassette, a double HA tag sequence and the terminator of the small subunit of the Rubisco from pea (*Pisum sativum*; accession no. X00806). pPWGTkan carries similar parts except that the HA tag is replaced by the enhanced GFP sequence.

### Western Blotting and Co-immunoprecipitation

Protein extraction and western blotting were performed as previously described, with the following modifications (Yu and Pilot, 2014): Leaves from each line (selected on kanamycin for 7 days, and transferred to soil and grown for three more weeks) were collected for protein extraction and western blot. Five hundred mg of leaves were ground with 1 ml of extraction buffer composed of 50 mM Tris-HCl, pH 7.3, 150 mM NaCl, 10 mM MgCl_2_, 10 mM DTT, 0.5% Nonidet P-40, and 1X Complete Protease Inhibitors (Roche) on ice. Homogenates were centrifuged at 14,000 *g* at 4°C for 15 min. Protein concentration of the supernatant was quantified by Bradford reagent. Twenty μg of total proteins were analyzed by SDS-PAGE (4–12% polyacrylamide MES gel; Life Technologies) and western blotting. Proteins were transferred on a nitrocellulose membrane (GE Healthcare) and detected using anti-HA (clone 3F10; Roche Diagnostics; 1:5,000) primary antibody, anti-rat (Thermo Scientific) secondary antibody, and the ECL-Prime western-blotting detection system (GE Healthcare). Co-immunoprecipitation experiments were performed from *N. benthamiana* infiltrated leaves as described (Pratelli et al., 2012).

### Amino Acid Uptake

Amino acid uptakes in plants were performed as described (Pratelli et al., 2010), from segregating T2 seeds selected on kanamycin before growing in liquid medium and uptake.

## Author Contributions

RP and GP designed and performed the suppressor screening. SY and RP characterized the suppressor mutants. SY and CD characterized the recapitulation lines and the mutagenized GDU1 proteins. SY and GP designed the experiments and wrote the paper.

## Conflict of Interest Statement

The authors declare that the research was conducted in the absence of any commercial or financial relationships that could be construed as a potential conflict of interest.

## References

[B1] Batoko H. Zheng H. Q. Hawes C. Moore I. (2000). A Rab1 GTPase is required for transport between the endoplasmic reticulum and golgi apparatus and for normal golgi movement in plants. *Plant Cell* 12 2201–2218. 10.1105/tpc.12.11.2201 11090219 PMC150168

[B2] Bonner C. A. Rodrigues A. Miller J. A. Jensen R. A. (1992). Amino acids are general growth inhibitors of *Nicotiana silvestris* in tissue culture. *Physiol. Plant.* 84 319–328. 10.1111/j.1399-3054.1992.tb04671.x

[B3] Chhangani D. Nukina N. Kurosawa M. Amanullah A. Joshi V. Upadhyay A. (2014). Mahogunin ring finger 1 suppresses misfolded polyglutamine aggregation and cytotoxicity. *Biochim. Biophys. Acta* 1842 1472–1484. 10.1016/j.bbadis.2014.04.014 24769000

[B4] Chivasa S. Ndimba B. K. Simon W. J. Lindsey K. Slabas A. R. (2005). Extracellular ATP functions as an endogenous external metabolite regulating plant cell viability. *Plant Cell* 17 3019–3034. 10.1105/tpc.105.036806 16199612 PMC1276027

[B5] Clough S. J. Bent A. F. (1998). Floral dip: a simplified method for *Agrobacterium*-mediated transformation of *Arabidopsis thaliana*. *Plant J.* 16 735–743. 10.1046/j.1365-313x.1998.00343.x 10069079

[B6] de Kraker J. W. Gershenzon J. (2011). From amino acid to glucosinolate biosynthesis: protein sequence changes in the evolution of methylthioalkylmalate synthase in *Arabidopsis*. *Plant Cell* 23 38–53. 10.1105/tpc.110.079269 21205930 PMC3051243

[B7] Dundar E. Bush D. R. (2009). BAT1, a bidirectional amino acid transporter in *Arabidopsis*. *Planta* 229 1047–1056. 10.1007/s00425-009-0892-8 19199104

[B8] Durek P. Schmidt R. Heazlewood J. L. Jones A. Maclean D. Nagel A. (2010). PhosPhAt: the *Arabidopsis thaliana* phosphorylation site database. An update. *Nucleic Acids Res.* 38 D828–D834. 10.1093/nar/gkp810 19880383 PMC2808987

[B9] Elbaz Y. Salomon T. Schuldiner S. (2008). Identification of a glycine motif required for packing in EmrE, a multidrug transporter from *Escherichia coli*. *J. Biol. Chem.* 283 12276–12283. 10.1074/jbc.M710338200 18321856 PMC2431008

[B10] Falcone Ferreyra M. L. Rius S. P. Casati P. (2012). Flavonoids: biosynthesis, biological functions, and biotechnological applications. *Front. Plant Sci.* 3:222 10.3389/fpls.2012.00222 PMC3460232 23060891

[B11] Guerra D. D. Pratelli R. Kraft E. Callis J. Pilot G. (2013). Functional conservation between mammalian MGRN1 and plant LOG2 ubiquitin ligases. *FEBS Lett.* 587 3400–3405. 10.1016/j.febslet.2013.08.045 24036454 PMC4157525

[B12] Gunn T. M. Silvius D. Bagher P. Sun K. Walker K. K. (2013). MGRN1-dependent pigment-type switching requires its ubiquitination activity but not its interaction with TSG101 or NEDD4. *Pigment Cell Melanoma Res.* 26 263–268. 10.1111/pcmr.12059 23253940 PMC4226269

[B13] Heazlewood J. L. Durek P. Hummel J. Selbig J. Weckwerth W. Walther D. (2008). PhosPhAt: a database of phosphorylation sites in *Arabidopsis thaliana* and a plant-specific phosphorylation site predictor. *Nucleic Acids Res.* 36 D1015–D1021. 10.1093/nar/gkm812 17984086 PMC2238998

[B14] Houck S. A. Cyr D. M. (2012). Mechanisms for quality control of misfolded transmembrane proteins. *Biochim. Biophys. Acta* 1818 1108–1114. 10.1016/j.bbamem.2011.11.007 22100602 PMC3288195

[B15] Jack D. L. Yang N. M. Saier M. H. Jr. (2001). The drug/metabolite transporter superfamily. *Eur. J. Biochem.* 268 3620–3639. 10.1046/j.1432-1327.2001.02265.x 11432728

[B16] Javadpour M. M. Eilers M. Groesbeek M. Smith S. O. (1999). Helix packing in polytopic membrane proteins: role of glycine in transmembrane helix association. *Biophys. J.* 77 1609–1618. 10.1016/S0006-3495(99)77009-8 10465772 PMC1300449

[B17] Jiao J. Sun K. Walker W. P. Bagher P. Cota C. D. Gunn T. M. (2009). Abnormal regulation of TSG101 in mice with spongiform neurodegeneration. *Biochim. Biophys. Acta* 1792 1027–1035. 10.1016/j.bbadis.2009.08.009 19703557 PMC2755232

[B18] Kim S. Jeon T. J. Oberai A. Yang D. Schmidt J. J. Bowie J. U. (2005). Transmembrane glycine zippers: physiological and pathological roles in membrane proteins. *Proc. Natl. Acad. Sci. U.S.A.* 102 14278–14283. 10.1073/pnas.0501234102 16179394 PMC1242278

[B19] Kunkel T. A. Bebenek K. Mcclary J. (1991). Efficient site-directed mutagenesis using uracil-containing DNA. *Methods Enzymol.* 204 125–139. 10.1016/0076-6879(91)04008-C 1943776

[B20] Ladwig F. Stahl M. Ludewig U. Hirner A. A. Hammes U. Z. Stadler R. (2012). Siliques are Red1 from *Arabidopsis* acts as a bidirectional amino acid transporter that is crucial for the amino acid homeostasis of siliques. *Plant Physiol.* 158 1643–1655. 10.1104/pp.111.192583 22312005 PMC3320175

[B21] Lalonde S. Sero A. Pratelli R. Pilot G. Chen J. Sardi M. I. (2010). A membrane protein/signaling protein interaction network for *Arabidopsis* version AMPv2. *Front. Physiol.* 1:24 10.3389/fphys.2010.00024 PMC3059934 21423366

[B22] Lee T. Y. Bretana N. A. Lu C. T. (2011). PlantPhos: using maximal dependence decomposition to identify plant phosphorylation sites with substrate site specificity. *BMC Bioinformatics* 12:261 10.1186/1471-2105-12-261 PMC3228547 21703007

[B23] Lee Y. H. Foster J. Chen J. Voll L. M. Weber A. P. Tegeder M. (2007). AAP1 transports uncharged amino acids into roots of *Arabidopsis*. *Plant J.* 50 305–319. 10.1111/j.1365-313X.2007.03045.x 17419840

[B24] Michaeli S. Fait A. Lagor K. Nunes-Nesi A. Grillich N. Yellin A. (2011). A mitochondrial GABA permease connects the GABA shunt and the TCA cycle and is essential for normal carbon metabolism. *Plant J.* 67 485–498. 10.1111/j.1365-313X.2011.04612.x 21501262

[B25] Nagy J. K. Sanders C. R. (2004). Destabilizing mutations promote membrane protein misfolding. *Biochemistry* 43 19–25. 10.1021/bi035918s 14705927

[B26] Ndimba B. K. Chivasa S. Hamilton J. M. Simon W. J. Slabas A. R. (2003). Proteomic analysis of changes in the extracellular matrix of *Arabidopsis* cell suspension cultures induced by fungal elicitors. *Proteomics* 3 1047–1059. 10.1002/pmic.200300413 12833529

[B27] Perez-Oliva A. B. Olivares C. Jimenez-Cervantes C. Garcia-Borron J. C. (2009). Mahogunin ring finger-1 (MGRN1) E3 ubiquitin ligase inhibits signaling from melanocortin receptor by competition with Galphas. *J. Biol. Chem.* 284 31714–31725. 10.1074/jbc.M109.028100 19737927 PMC2797242

[B28] Pilot G. Stransky H. Bushey D. F. Pratelli R. Ludewig U. Wingate V. P. (2004). Overexpression of GLUTAMINE DUMPER1 leads to hypersecretion of glutamine from hydathodes of *Arabidopsis* leaves. *Plant Cell* 16 1827–1840. 10.1105/tpc.021642 15208395 PMC514164

[B29] Pratelli R. Guerra D. D. Yu S. Wogulis M. Kraft E. Frommer W. B. (2012). The ubiquitin E3 ligase LOSS OF GDU2 is required for GLUTAMINE DUMPER1-induced amino acid secretion in *Arabidopsis*. *Plant Physiol.* 158 1628–1642. 10.1104/pp.111.191965 22291198 PMC3320174

[B30] Pratelli R. Pilot G. (2006). The plant-specific VIMAG domain of Glutamine Dumper1 is necessary for the function of the protein in *Arabidopsis*. *FEBS Lett.* 580 6961–6966. 10.1016/j.febslet.2006.11.064 17157837

[B31] Pratelli R. Pilot G. (2007). Altered amino acid metabolism in glutamine dumper1 plants. *Plant Signal. Behav.* 2 182–184. 10.4161/psb.2.3.3972 19704691 PMC2634052

[B32] Pratelli R. Voll L. M. Horst R. J. Frommer W. B. Pilot G. (2010). Stimulation of nonselective amino acid export by glutamine dumper proteins. *Plant Physiol.* 152 762–773. 10.1104/pp.109.151746 20018597 PMC2815850

[B33] Ranocha P. Dima O. Nagy R. Felten J. Corratge-Faillie C. Novak O. (2013). *Arabidopsis* WAT1 is a vacuolar auxin transport facilitator required for auxin homoeostasis. *Nat. Commun.* 4:2625 10.1038/ncomms3625 PMC3826630 24129639

[B34] Rosnoblet C. Legrand D. Demaegd D. Hacine-Gherbi H. De Bettignies G. Bammens R. (2013). Impact of disease-causing mutations on TMEM165 subcellular localization, a recently identified protein involved in CDG-II. *Hum. Mol. Genet.* 22 2914–2928. 10.1093/hmg/ddt146 23575229

[B35] Snowden C. J. Thomas B. Baxter C. J. Smith J. A. Sweetlove L. J. (2015). A tonoplast Glu/Asp/GABA exchanger that affects tomato fruit amino acid composition. *Plant J.* 81 651–660. 10.1111/tpj.12766 25602029 PMC4950293

[B36] Tegeder M. (2014). Transporters involved in source to sink partitioning of amino acids and ureides: opportunities for crop improvement. *J. Exp. Bot.* 65 1865–1878. 10.1093/jxb/eru012 24489071

[B37] Tegeder M. Rentsch D. (2010). Uptake and partitioning of amino acids and peptides. *Mol. Plant* 3 997–1011. 10.1093/mp/ssq047 21081651

[B38] Trainer M. A. Yurgel S. N. Kahn M. L. (2007). Role of a conserved membrane glycine residue in a dicarboxylate transporter from *Sinorhizobium meliloti*. *J. Bacteriol.* 189 2160–2163. 10.1128/JB.01247-06 17158675 PMC1855765

[B39] Vastermark A. Wollwage S. Houle M. E. Rio R. Saier M. H. Jr. (2014). Expansion of the APC superfamily of secondary carriers. *Proteins* 82 2797–2811. 10.1002/prot.24643 25043943 PMC4177346

[B40] Voinnet O. Rivas S. Mestre P. Baulcombe D. (2003). An enhanced transient expression system in plants based on suppression of gene silencing by the p19 protein of tomato bushy stunt virus. *Plant J.* 33 949–956. 10.1046/j.1365-313X.2003.01676.x 12609035

[B41] Yang H. Bogner M. Stierhof Y. D. Ludewig U. (2010). H^+^-independent glutamine transport in plant root tips. *PLoS ONE* 5:e8917 10.1371/journal.pone.0008917 PMC2811748 20111724

[B42] Yoo B. C. Kragler F. Varkonyi-Gasic E. Haywood V. Archer-Evans S. Lee Y. M. (2004). A systemic small RNA signaling system in plants. *Plant Cell* 16 1979–2000. 10.1105/tpc.104.023614 15258266 PMC519190

[B43] Yu S. Pilot G. (2014). Testing the efficiency of plant artificial microRNAs by transient expression in *Nicotiana benthamiana* reveals additional action at the translational level. *Front. Plant Sci.* 5:622 10.3389/fpls.2014.00622 PMC4237044 25477887

[B44] Ziegler J. Facchini P. J. (2008). Alkaloid biosynthesis: metabolism and trafficking. *Ann. Rev. Plant Biol.* 59 735–769. 10.1146/annurev.arplant.59.032607.092730 18251710

