# Supplementary material for: Suppressor mutations in the Glutamine Dumper1 protein dissociate disturbance in amino acid transport from other characteristics of the Gdu1D phenotype
Source: Front Plant Sci. 2015 Aug 4;6:593. doi: 10.3389/fpls.2015.00593 (PMC4523740; doi:10.3389/fpls.2015.00593)
Supplement: Supplementary file 1 [file Presentation_1.PDF]

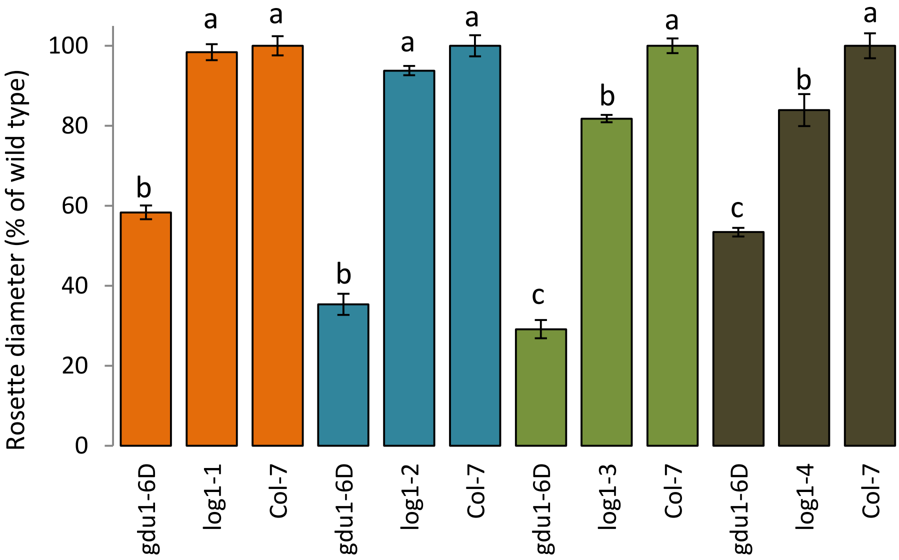

Figure S1. Size analysis of the *log1* suppressor mutants.
Plants were grown on soil and the diameter of the rosette was measured at bolting time. Each mutant was analyzed in a different batch (shown by different colors), for which the size of the parental line (*gdu1-6D*) and the wild type (Col-7; set at 100%) was recorded. Error bars = SEM (n = 20 to 33 plants); statistical significance determined by ANOVA using Tukey's HSD, $p<0.01$ (performed for each batch).

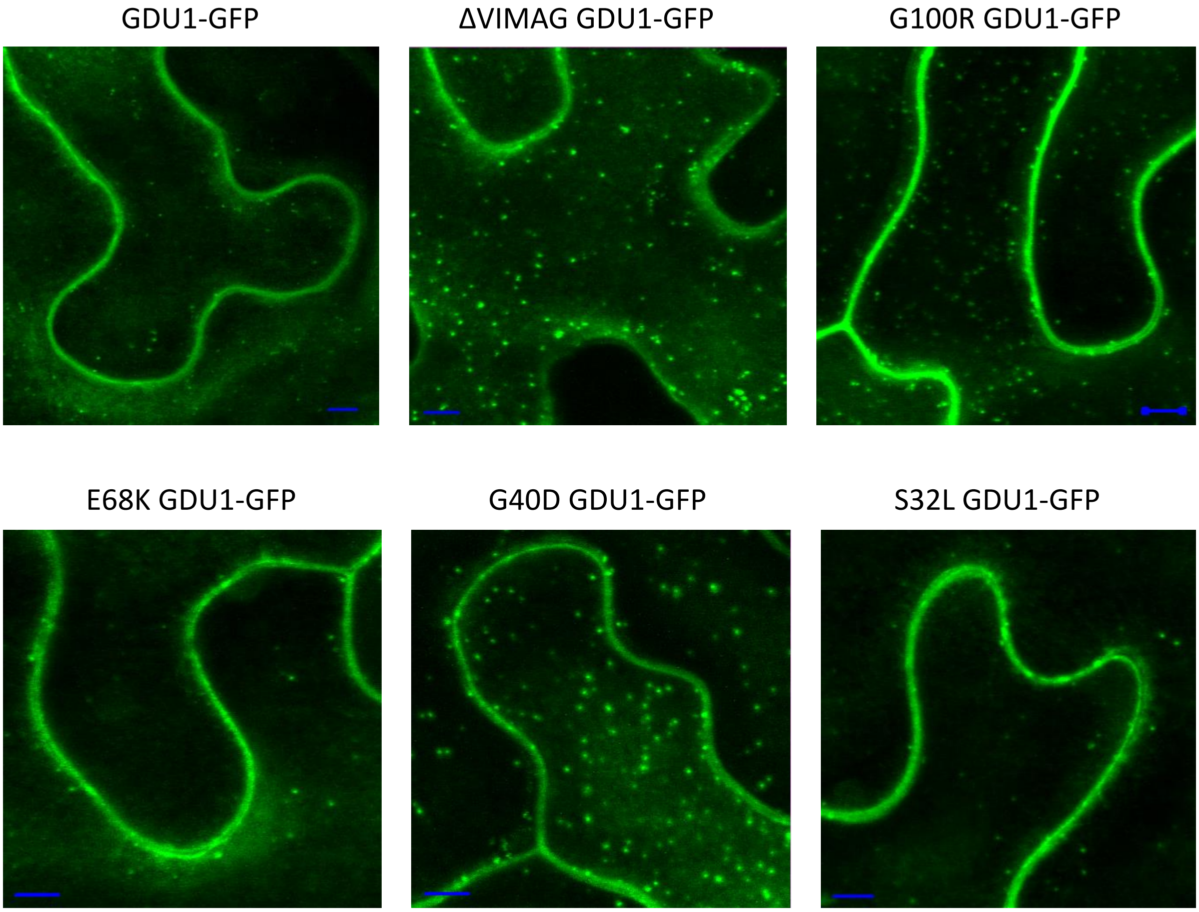

Figure S2. Sub-cellular localization of the GDU1 variants in *N. benthamiana* cells.
Constructs leading to the fusion of the GDU1 variants under the control of the 35S promoter and in fusion with the GFP were transiently expressed in *N. Benthamiana* epidermis cells by agro infiltration. Pictures shown correspond to maximum intensity computed from a z-stack of 24-35 images of the tangential side of cells. Pictures were edited in Adobe Photoshop CS5 using the auto tone tool. Blue bar = 5 µm. Imaging was performed as previously described (Pratelli, R., Guerra, D.D., Yu, S., Wogulis, M., Kraft, E., Frommer, W.B., Callis, J., and Pilot, G. (2012). The ubiquitin E3 ligase LOSS OF GDU2 is required for GLUTAMINE DUMPER1-induced amino acid secretion in Arabidopsis. Plant Physiol 158, 1628-1642).

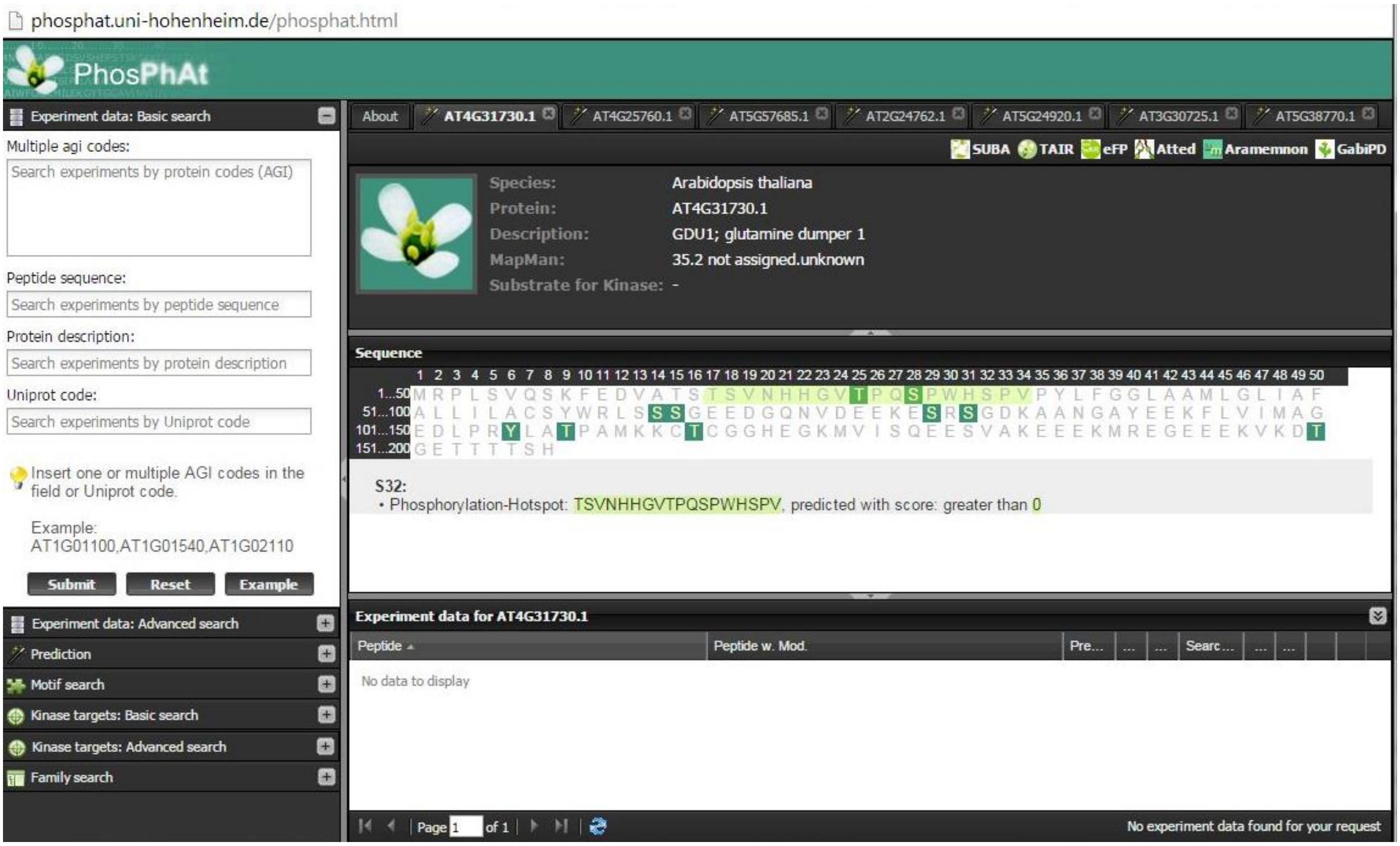

Figure S3. GDU1 phosphorylation sites predicted by PhosPhAt 4.0. Screen capture from http://phosphat.uni-hohenheim.de/.

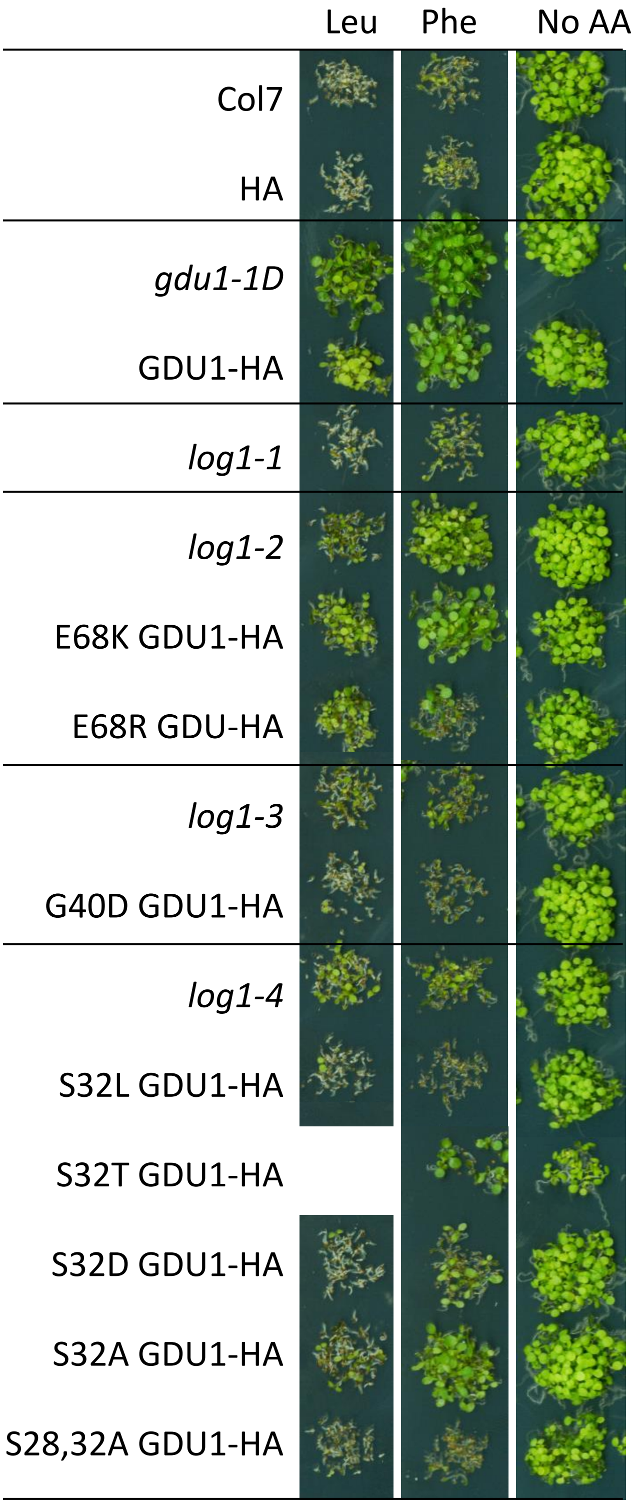

Figure S4. Amino acid tolerance of the *log1* suppressor mutants and of lines over-expressing the *GDU1* variants. Plants were grown for 10 days in long day conditions, on half-strength MS medium, supplemented with 0.5% sucrose, and 10 mM of amino acids, where indicated. The S32T GDU1-HA line was not sown on the Leu supplemented medium in this experiment.

Name	Oligo Sequence	Purpose
GDU1 S28,32A	GTGACGCCGCAAGCTCCGTGGCACGCTCCGGTTCCTTACCTC	Mutagenesis
GDU1 S32T	TCACCGTGGCACACTCCGGTTCCTTACCTC	Mutagenesis
GDU1 S32A	TCACCGTGGCACGCTCCGGTTCCTTACCTC	Mutagenesis
GDU1 S32D	TCACCGTGGCACGATCCGGTTCCTTACCTC	Mutagenesis
GDU1 E68R	TCCTCCGGCGAAAGAGATGGTCAAAACGTA	Mutagenesis
GDU1 attB1	GGGGACAAGTTTGTACAAAAAAGCAGGCTTAATGAGACCATTGAGCGTA	Gateway cloning
GDU1 attB2	GGGGACCACTTTGTACAAGAAAGCTGGGTAGTGACTTGTAGTAGTTGTCT	Gateway cloning

Table S1. Sequence of the oligonucleotides used in this study.
